# The medical home functions of primary care practices that care for adults with psychological distress: a cross-sectional study

**DOI:** 10.1186/s12913-018-3845-8

**Published:** 2019-01-09

**Authors:** Shawn Linman, Ivy Benjenk, Jie Chen

**Affiliations:** School of Public Health, University of Maryland, 4200 Valley Dr #2242, College Park, MD 20742 USA

**Keywords:** Mental health, Medical home, Primary care

## Abstract

**Background:**

Primary care practices are changing the way that they provide care by increasing their medical home functionality. Medical home functionality can improve access to care and increase patient-centeredness, which is essential for persons with mental health issues. This study aims to explore the degree to which medical home functions have been implemented by primary care practices that care for adults with psychological distress.

**Methods:**

Analysis of the 2015 Medical Expenditure Panel Survey Household Component and Medical Organizations Survey. This unique data set links data from a nationally representative sample of US households to the practices in which they receive primary care. This study focused on adults aged 18 and above.

**Results:**

As compared to adults without psychological distress, adults with psychological distress had significantly higher rates of chronic illness and poverty. Adults with psychological distress were more likely to receive care from practices that include advanced practitioners and are non-profit or hospital-based. Multivariate models that were adjusted for patient-level and practice-level characteristics indicated that adults with psychological distress are as likely to receive primary care from practices with medical home functionality, including case management, electronic health records, flexible scheduling, and PCMH certification, as adults without psychological distress.

**Conclusions:**

Practices that care for adults with mental health issues have not been left behind in the transition towards medical home models of primary care. Policy makers should continue to prioritize adults with mental health issues to receive primary care through this model of delivery due to this population’s great potential to benefit from improved access and care coordination.

**Trial registration:**

This study does not report the results of a health care intervention on human subject’s participants.

## Background

Adults with poor mental health or psychological distress (PD) have complex primary care needs. Primary care physicians are often the only mental health providers for adults with common mental health conditions, like anxiety and depression [1, 2]. For these patients, primary care physicians are responsible for diagnosing and managing both physical and mental health conditions. Adults with severe mental illnesses (SMI), like schizophrenia and bipolar disorder, are at significantly higher risk of having diabetes, cardiovascular diseases, and chronic respiratory diseases than the general adult population [3–6]. Adults with PD experience psychosocial and economic barriers that make it difficult to follow treatment recommendations, including unstable housing, unpredictable schedules, lack of transportation, limited social supports, and inability to afford copayments [7–9]. Adults with PD also have complex care coordination needs; they may receive various services from community mental health clinics and substance abuse treatment programs, which have long been siloed from medical care providers [10]. Primary care providers struggle to sufficiently care for patients with multiple medical and psychiatric comorbidities in setting of socioeconomic challenges and care coordination needs under the constraints of the traditional fifteen minute office visit [11, 12].

Furthermore, adults with PD experience numerous barriers to accessing primary care and receiving patient-centered primary care, even when they have regular primary care physicians. Adults with PD have reported experiencing anxiety and paranoia while waiting for long periods of time in crowded waiting rooms or alone in examination rooms [11, 13] and have been found to be less likely to receive a same-day response when they contact their primary care practice for a medical issue [14]. They have reported difficulty developing trusting relationships with primary care providers and feeling like their providers do not understand their concerns [15]. Nearly 15% of adults with SMI who have primary care physicians report that they still use the emergency department as their main source of routine care [16]. These barriers to primary care utilization may contribute to the high rate of potentially preventable hospitalizations in this population [17].

Recent studies have shown that new models of primary care may be more effective for persons with PD. These new models of care rely on flexible access to primary care services, a primary care team that includes case managers, and technologies that continue the patient-provider relationship outside of the office visit [18, 19]. Adults with depression and poorly-controlled chronic illness have been found to have greater improvement in glycemic control, cholesterol levels, systolic blood pressure, and depression scores when they receive primary care from practices where nurse case managers meet with patients regularly and monitor mood symptoms, medical diseases, self-care activities, and medical adherence [20, 21]. Veterans with posttraumatic stress disorder have been found to have higher rates of primary care utilization and lower rates of specialty care utilization and hospitalization when they receive primary care from practices that reduce barriers to care by offering same day appointments, telephone appointments, and electronic messaging as well as track quality metrics, provide care through multidisciplinary teams, and assign care coordinators to high-risk patients [22]. Adults with chronic medical conditions and comorbid mental health conditions who receive care from primary care practices that are certified as patient-centered medical homes (PCMHs) have been found to lower expected mean counts of emergency department (ED) visits and psychiatric hospitalizations [23].

PCMHs or medical homes are sites of comprehensive primary care that optimize patient-centeredness, access, timeliness, care coordination, treatment planning, patient and caregiver engagement, and population health [24, 25]. Medical homes offer same-day and non-business hours appointments to reduce barriers to accessing primary care. They use care coordinators, registries, and electronic decision support to ensure that all patients in the practice receive appropriate screenings and preventative services. Medical homes also use team-based models of care delivery in order to maximize time that providers are able to spend with patients [18, 19]. The medical home model has been supported by a number of medical professional societies and is being encouraged by the federal government under the alternative payment model provisions of the Patient Protection and Affordable Care Act (PPACA) and the Medicare Access and CHIP Reauthorization Act (MACRA) [26, 27]. This model is specifically encouraged for adults with SMI or common mental illness and comorbid chronic medical conditions under Medicaid Health Home programs [28]. Primary care practices that have many of these medical home functions have generally been found to have higher rates of patient satisfaction, staff satisfaction, and preventative care utilization and lower rates of ED utilization and potentially preventable hospitalizations [29–32].

The objective of this study was to examine whether adults with PD are as likely to receive primary care from practices that have implemented the medical home functions as adults without PD. Previous work has shown variation in adoption of these functions across primary care practices [33]. Patients who are uninsured and live in the South have been found to be less likely to receive care from practices with medical home functions [34]. We were interested in determining if persons with psychological distress are less likely to receive primary care from practices with the medical home functions than persons without psychological distress, because previous literature has found that adults with SMI are less likely to receive high quality medical care [35]. Persons with psychological distress are also more likely to be low-income and publicly insured than persons without psychological distress. Low income patients have previously been found to receive care from physicians with lower quality ratings and to report greater ease accessing care at the emergency room than from their primary care physicians [36, 37].

## Methods

### Data source and analytic sample

We used data from the 2015 Medical Expenditure Panel Survey (MEPS) Household Component and MEPS Medical Organization Survey. The MEPS Household Component is nationally representative survey of non-institutionalized citizens in the United States. It is conducted by the Agency for Healthcare Research and Quality (AHRQ). The MEPS Household Component surveys households on demographics, socioeconomic status, perceptions of health and healthcare, healthcare experiences, healthcare utilization, healthcare spending, health outcomes, and insurance status [38]. As part of the household survey, a sample of medical provider practices (Medical Provider Component or MPC) that cared for the survey participants were contacted by telephone and asked to provide information about charges, payments, diagnoses, and procedures.

In 2015, AHRQ added a Medical Organizations Survey (MOS) component to the survey with the purpose of understanding the organizational and financial characteristics of the practices where household survey participants receive their primary care [39]. The MEPS asked the 11,188 household survey participants who reported having a usual care provider and seeing that usual care provider within the last year if they would be willing to participate in the MOS component. The usual care provider was identified by MEPS during the MPC as the primary location of the participants office-based care. MEPS contacted each practice to determine an appropriate respondent [40].

9494 MEPS household survey participants agreed to participate and 4318 provider practices serving 7350 MEPS household participants agreed to participate, as more than one MEPS household participant may receive care at the same practice. The MOS was predominantly administered via telephone (92.9%) by data collection specialists and completed by office managers and practice administrators [34, 40]. The MEPS MOS respondents were asked questions about the organizational characteristics and medical home functionality of their practices. For example, the MOS survey included the following question: “Is the electronic records system routinely used for exchanging secure messages with patients?”

Of the 7350 household survey participants with corresponding MOS responses, we restricted our analysis to participants age 18 and above (*n* = 4632). We dropped observations with missing K6 scores (7%), resulting in a final sample of 4290 household survey participants, representing 67,745,443 Americans. We found that participants were missing one or more covariates at a rate of 14.2% among participants with PD and 12.5% among participants without PD. We compared populations with and without PD that were missing one or more practice covariates and found a significant difference in implementation of EHR reminders (coefficient = 0.474, *p =* 0.026) among the PD and non-PD missing populations.

We linked the MOS practice responses to the MEPS household survey participant responses at the level of the individual MEPS household survey participant. We structured all variables from the perspective of the household survey participant in an attempt to understand the medical home functionality of the sites in which participants receive primary care.

### Ethics

As we used publicly available de-identified survey data collected by the federally government, we did not have to consent participants for participation or publication and ethics approval was not required.

### Variables

#### Medical home functions

For our initial research question, we evaluated implementation of medical home functions at the participant’s site of usual care as our outcome variables. These medical home functions included: utilization of case managers to coordinate care, adoption of EHRs with secure messaging and guideline reminders, timely follow-up appointments after hospitalization, same-day appointment availability, preventative care and follow-up reminders for patients, multi-specialty integration, personalized quality reports for providers, and certification as a Patient-Centered Medical Home (PCMH) by a certifying body [41].

#### Psychological distress

Our main independent variable was psychological distress as measured by the Kessler Psychological distress (K6) screening assessment embedded in the MEPS survey. The K6 consists of six questions that ask participants to rate how often they felt nervous, hopeless, restless or fidgety, intensely depressed, unable to complete tasks, and worthless during the past month and during the worst month that they had in the past year [42]. The K6 has been found to be useful broad screening tool for mental health conditions and has been identified as an effective method for screening for mental disorders during surveys [43]. Our study defines psychological distress as a K6 score of thirteen or greater [43].

#### Other covariates

We used the Andersen social behavioral model [44] and earlier work by Levine and colleagues [34] using the MEPS MOS to select patient-level covariates to include in our analysis. The patient-level covariates in our study can be categorized into three domains: the predisposing factors (race/ethnicity, age, gender, marital status, and interview language); enabling factors (education, insurance status, income); and clinical needs factors (perceived health status, physical disability, obesity, smoker status, and medical comorbidities). We also controlled for the following practice-level characteristics: total number of providers (physicians and advanced practitioners), ownership status (physician-owned, hospital-owned, other-owned), and multiple locations [45].

### Statistical analysis

We first used descriptive statistics to compare the population characteristics and primary care practice characteristics for the PD and non-PD populations and considered any value of *p* < 0.05 to be significant. In order to examine whether adults with PD are more or less likely to be cared for by practices that have implemented the medical home functions than adults without PD, we performed separate multivariate logistic regression analyses to obtain odds ratios for each medical home function.

We also created a composite outcome variable of the total number of medical home functions in place at the primary care practice (i.e. the summation of EHR reminders/guidelines, exchanges secure messages, case manager, 48-hour post-discharge communication, preventative care reminders for patients, same-day appointments, multispecialty practice, and personalized quality reports for physicians). We did not include EHR implementation and PCMH certification in this composite due to the overlap with the other functions. Hence, this composite measure was an additive value ranging from 0 to 8 dependent on the number of medical home functions utilized by a practice. We then performed a linear regression with the composite variable as our main outcome of interest. We adjusted for patient-level and provider-level characteristics in both sets of regressions.

We performed all analyses with STATA 15. In order to account for complex survey design features employed in the MEPS, including clustering and stratification at the household level, we used the survey suite of commands in STATA. We applied survey weights that are unique to the MOS component to make our dataset nationally representative. We also tested the robustness of our finding using various sensitivity analyses, including different composite measures and model specifications. Results were all similar and are available upon request.

## Results

Table 1 presents the population characteristics of our linked data set. We found that while 6.22% (*n* = 267) of participants in our study had a K6 score of 13 or greater indicating PD, only 4.6% of participants in the MEPS household survey met this criterion.

**Table 1:** Selected characteristics of the adult US civilian, non-institutionalized populations based on K-6 screening for psychological distress (PD), MEPS 2015

Characteristic	Patients with PD Diagnosis	Patients with no PD Diagnosis
	Mean % (95% CI)^a^ (*n* = 267)	Mean % (95% CI)^†^ (*n* = 4023)
Age, in years	52 (48, 55)	53 (52, 54)
Female	64 (55, 72)	58 (56, 60)
Race/Ethnicity		
White Non-Hispanic	65 (56, 73)	68 (65, 71)
Black Non-Hispanic	9 (6, 13)	10 (9, 12)
Hispanic	19 (13, 26)	14 (12, 16)
Other Non-Hispanic	7 (3, 15)	8 (6, 11)
Marital status		
Married	71 (63, 80)	78 (76, 80)
Never Married	28 (20, 37)	22 (20, 24)
Education		
No High School Degree	12 (8, 18)	5 (4, 6)
High School Degree or Above	88 (82, 92)	95 (94, 96)
Insurance status		
Any Private	32 (24, 42)	69 (67, 71)
Public Only	61 (50, 71)	26 (24, 28)
Uninsured	7 (4, 12)	5 (3, 6)
Income		
Low Income	65 (58, 73)	29 (27, 31)
Middle income	24 (17, 32)	29 (27, 32)
High income	10 (6, 18)	42 (39, 45)
Interview language		
English	93 (92, 94)	89 (82, 93)
Other	7 (5, 8)	11 (6, 18)
Percieved Health Status		
Excellent/Very Good	13 (7, 22)	51 (49, 53)
Good/Fair/Poor	87 (78, 93)	49 (47, 51)
Chronic Conditions		
No Chronic Conditions	14 (10, 20)	30 (28, 33)
High Blood Pressure	62 (53, 70)	46 (44, 49)
Diabetes	29 (22, 38)	15 (13, 16)
Cancer	15 (10, 23)	15 (13, 16)
Coronary Heart Disease	13 (9, 19)	8 (6, 9)
High Cholesterol	50 (42, 59)	43 (41, 46)
Asthma	26 (19, 35)	12 (11,13)
Stroke	16 (9, 25)	5 (5, 6)
Angia	7 (4, 12)	4 (3, 5)
Heart Attack	13 (8, 19)	5 (5, 6)
Census Region		
Northeast	12 (9, 17)	19 (16, 22)
Midwest	22 (17, 28)	21 (18, 24)
South	44 (35, 52)	36 (32, 39)
West	22 (17, 29)	25 (22, 28)

Data from MEPS, 2015 household component and medical organization survey files

Data was weighted to adjust for complex survey design and is nationally representative

^a^May not sum to 100 due to rounding

Abbreviations: MEPS is the Medical Expenditure Panel Survey

As compared to household survey participants without PD, a significantly greater percentage of participants with PD had medical conditions or risk factors that require close primary care management, including hypertension (62% vs. 46%), diabetes (29% vs. 15%), coronary heart disease (13% vs. 8%), stroke (16% vs. 5%), myocardial infarction (13% vs. 5%), asthma (26% vs. 12%), and fair or poor perceived health status (87% vs. 49%). In contrast, a significantly lower percentage of participants with psychological distress had enabling factors for primary care engagement and compliance with follow-up recommendations, including marriage (71% vs. 78%), a high school diploma (88% vs. 95%), and income above 400% of the federal poverty line (10% vs. 42%).

Table 2 presents the different characteristics between primary care practices that care for adults with and without PD. A significantly greater percentage of participants with PD received their primary care from nonprofit or hospital-owned practices (46% vs. 34%), while a significantly smaller percentage received their care from physician-owned primary care practices (45% vs. 59%). A significantly greater percentage of participants with PD received care from practices that utilize nurse practitioners and physician assistants (79% vs. 72%). There were no statistical differences between the percentage of participants with and without PD that receive their primary care from practices that have multiple locations.

**Table 2:** Selected characteristics of primary care practices based on interviewee’s psychological distress (PD) screening, MEPS 2015

	Patients with PD Diagnosis	Patients with no PD Diagnosis
Characteristic	Mean % (95% CI)^†^	Mean % (95% CI)^a^
Practice Ownership		
Physician-owned	45 (37, 54)	59 (56, 61)
Hospital-owned	46 (38, 54)	34 (31, 36)
Other-owned	9 (5, 15)	8 (6, 9)
Multiple Locations	48 (39, 58)	44 (42, 47)
Multispecialty Practice	45 (36, 55)	40 (38, 43)
Prescribers Per Practice		
1	12 (7, 19)	14 (13, 16)
2–10	56 (47, 65)	56 (54, 58)
> 10	32 (24, 42)	30 (27, 32)
Uses advanced practitioners	79 (71, 86)	72 (70, 74)
Uses EHR	92 (86, 96)	91 (89, 93)
EHR Reminders/Guideline for providers	93 (88, 96)	89 (87, 91)
Exchanges Secure Messages	75 (65, 83)	81 (79, 83)
PCMH Certification	47 (37, 57)	48 (45, 51)
Case Mananger	50 (41, 59)	52 (49, 54)
Contact within 48-hours of discharge	75 (66, 82)	77 (74, 79)
Preventative care reminders for patients	92 (87, 96)	89 (87, 91)
Same day appointments	94 (89, 96)	94 (93, 94)
Provider-level quality reports	94 (90, 97)	89 (88, 91)

Data from MEPS, 2015 household component and medical organization survey files

Data was weighted to adjust for complex survey design and is nationally representative

^a^May not sum to 100 due to rounding

Although we found that participants with PD receive care from different types of practices than participants without PD, we did not find much difference between the two groups as related to the medical home functionality of their site of primary care. We found no significant between-group differences in the implementation of electronic health records (92% vs. 91%), secure messaging with patients (75% vs. 81%), same-day appointment availability (94% vs. 94%), follow-up appointments within forty-eight hours of hospitalization (75% vs. 77%), preventative care reminders (92% vs. 89%), or case managers (50% vs. 52%). We also found no differences in multi-specialty practice (45% vs. 40%) and PCMH certification (47% vs. 48%). However, we did find that a significantly greater percentage of participants with PD receive their primary care from practices where physicians routinely receive quality reports (94% vs. 89%, *p* = 0.003) and that utilize clinical decision support technologies through their electronic health record systems (93% vs 89%, *p* = 0.021).

Table 3 shows the results of our multivariable linear regression using the composite measure of eight medical home functions. Our regression model found that there is no significant difference between participants with PD and participants without PD in their likelihood to be seen by a practice that has implemented medical home functions (Coefficient = − 0.206, *p* = 0.141). This model did show that hospital-owned or non-profit practices (Coefficient = − 0.486, *p* < 0.001), practices with 2–10 or 11 prescribers (Coefficients = 0.721, 1.298, *p* < 0.001 for both outcomes), and practices with multiple locations (Coefficient = 0.272, *p* = 0.001) are more likely to have implemented the medical home functions. We also found that practices in southern states were significantly less likely to implement medical home functions than practices in northeastern states (Coefficient = − 0.260, *p* = 0.018).

**Table 3:** Results of linear regression using an additive composite index of patient-centered variables based on interviewee’s psychological distress (PD) screening

Variables	Coefficients	*P*-Values	95% Confidence Intervals	
Negative PD SCREEN	(reference)	(reference)	(reference)	(reference)
POSITIVE PD SCREEN	− 0.206	0.141	− 0.481	0.069
18–24	(reference)	(reference)	(reference)	(reference)
25–44	0.150	0.411	−0.209	0.509
45–64	0.259	0.160	−0.103	0.621
65+	0.362	0.075	−0.037	0.761
MALE	(reference)	(reference)	(reference)	(reference)
FEMALE	−0.019	0.765	−0.142	0.104
WHITE NON-HISPANIC	(reference)	(reference)	(reference)	(reference)
BLACK NON-HISPANIC	0.039	0.736	−0.188	0.265
OTHER NON-HISPANIC	−0.133	0.446	−0.476	0.211
HISPANIC	−0.005	0.968	−0.252	0.242
MARRIED	(reference)	(reference)	(reference)	(reference)
NEVER MARRIED	0.156	0.084	−0.021	0.333
NO HIGH SCHOOL DEGREE	(reference)	(reference)	(reference)	(reference)
High School DEGREE OR ABOVE	0.018	0.855	−0.172	0.207
Any Private Insurance	(reference)	(reference)	(reference)	(reference)
Any Public Insurance	−0.139	0.163	−0.334	0.057
UNINSURED	−0.288	0.086	−0.617	0.041
Excellent Health Status	(reference)	(reference)	(reference)	(reference)
Good/Very Good Health	0.115	0.207	−0.064	0.293
Fair/Poor Health Status	0.101	0.309	−0.095	0.298
LOW INCOME	(reference)	(reference)	(reference)	(reference)
MIDDLE INCOME	0.107	0.275	−0.086	0.300
HIGH INCOME	0.060	0.550	−0.137	0.256
ENGLISH	(reference)	(reference)	(reference)	(reference)
OTHER	0.257	0.076	−0.028	0.541
NO CHRONIC CONDITION	(reference)	(reference)	(reference)	(reference)
ONE OR MORE CHRONIC CONDITIONS	0.082	0.242	−0.056	0.219
EAST	(reference)	(reference)	(reference)	(reference)
MIDWEST	−0.092	0.453	−0.333	0.149
SOUTH	−0.260	0.018*	−0.474	−0.046
WEST	−0.096	0.446	−0.345	0.153
PHYSICIAN-OWNED	(reference)	(reference)	(reference)	(reference)
NONPROFIT/HOSPITAL-OWNED	0.486	< 0.001***	0.287	0.685
OTHER-OWNED	0.217	0.197	−0.113	0.548
1 PRESCRIBER	(reference)	(reference)	(reference)	(reference)
2–10 PRESCRIBERS	0.721	< 0.001***	0.457	0.985
11+ PRESCRIBERS	1.298	< 0.001***	1.004	1.591
PRACTICE HAS ONE LOCATION	(reference)	(reference)	(reference)	(reference)
PRACTICE HAS MULTIPLE LOCATIONS	0.272	0.001**	0.109	0.435
CONSTANT	4.841	< 0.001***	4.373	5.309

Data from MEPS, 2015 household component and medical organization survey files

Data was weighted to adjust for complex survey design and is nationally representative

*p < 0.05, **p < 0.01, ***p < 0.001

R-squared = 0.1809

Table 4 displays the final results of the multivariate logistic regressions for each of the ten medical home functions that we included in our analysis. After controlling for patient-level predisposing, enabling, and need factors, as well as provider-level characteristics, we found the participants with PD had no greater or less likelihood of receiving primary care from practices that have implemented any of the medical home functions as compared with participants without PD.

**Table 4:** Results of multivariate regression models using individual measures of patient-centered practices based on interviewee’s screening for psychological distress (PD)

Patient-Centered Measures	Model 1 (Controlling for patient characteristics)				Model 2 (Controlling for patient andprovider characteristics)			
	Coefficients	*p*- Values	95% Confidence Intervals		Coefficients	*p*- Value	95% Confidence Intervals	
Uses EHR	0.313	0.295	−0.274	0.899	0.291	0.405	−0.397	0.979
EHR reminders/guidelines for providers	0.504	0.090	−0.079	1.087	0.468	0.128	−0.135	1.071
Exchanges secure messages with patients	−0.192	0.335	−0.583	0.200	−0.238	0.282	−0.672	0.197
PCMH certification	−0.102	0.580	−0.463	0.260	−0.216	0.245	−0.581	0.149
Case manager	−0.106	0.517	−0.428	0.216	−0.284	0.092	−0.615	0.047
Contact within 48-hours of discharge	−0.078	0.705	−0.483	0.327	−0.125	0.571	−0.560	0.309
Preventative care reminders for patients	0.413	0.226	−0.258	1.084	0.304	0.392	−0.395	1.002
Same day appointments	−0.131	0.692	−0.783	0.521	0.033	0.925	−0.653	0.719
Multispecialty practice	0.158	0.354	−0.178	0.495	0.105	0.599	−0.287	0.496
Provider-level quality reports	0.563	0.057	−0.017	1.143	0.410	0.206	−0.228	1.048

Data from MEPS, 2015 household component and medical organization survey files.

Data was weighted to adjust for complex survey design and is nationally representative.

*p < 0.05, **p < 0.01, ***p < 0.001

## Discussion

We are encouraged by the high levels of adoption of medial home functions by primary care practices participating in the 2015 MEPS MOS component. Nearly 90% of practices reported offering same day appointments, electronic health records with clinical decision support, and individualized physician quality reports. Nearly half of practices reported using care coordinators and having PCMH certification. We did also not find any disparities as they relate to the type of patients that receive care from practices with the medical home functions; we did not find any differences by age, gender, race, insurance type, socioeconomic status, and English language proficiency.

However, our results showed regional variation in the adoption of medical home functions. We found the South was significantly less likely to adopt medical home functions than the North, and there was no difference between the West, the Midwest, and the Northeast. This finding aligns with state-level legislation promoting medical home functionality in primary care practices. Patient-Centered Primary Care Collaborative initiatives indicated that all states in the Northeast have a law promoting medical home functionality, while very few states in the South have state-level law [46]. The results of this failure to adopt medical home functions in the South may be contributing to delays in care as persons with PD in the South have been found to be more likely to delay care than persons with PD living in other regions. This is especially notable because the highest proportion of white and black persons with PD live in the South [47]. Results of our study suggest the importance of regulation to promote the adoption of medical home functions. Future research should further investigate the effectiveness of these laws in terms of promoting health care access and quality.

We are also encouraged by the finding that adults with PD receive their primary care at practices that have implemented medical homes functions to the same degree as adults without PD. Due to their complex medical and psychosocial comorbidities, adults with mental health issues have significant potential to benefit from primary care that is delivered through a medical home. Furthermore, we found that a greater proportion of adults with PD are cared for in practices that have structures that align with the medical home model, including the use of advanced practitioners, which fosters a team-based approach, and participation in risk-bearing payment contracts, which increases provider accountability.

We also found that adults with PD are somewhat more likely to receive care in practices with two of the medical home functions: use of EHR clinical reminders and individual quality reports for physicians. However, these differences disappeared when controlling for patient-level and practice-level characteristics. These differences in medical home adoption are likely due to differences in the characteristics of medical practices that provide care to adults with PD; adults with PD are more likely to receive care from larger practices and non-profit or hospital-based practices and these practices are also more likely to have implemented the medical home functions. One could posit that practices that are owned by physicians would be less likely to implement clinical practice reminders in the EHR and personalized physician quality reports as these functions could be interpreted as threats to physician autonomy [48]. Smaller practices may also lack the infrastructure and health information technologies to provide personalized quality reports [49, 50].

Persons with mental illness have historically received lower quality medical care. We are very encouraged that although persons with PD receive care in practices with different organizational characteristics than persons without PD, they have not been left behind in this movement towards medical homes. The MOS survey did not assesses whether the practices surveyed were Federally Qualified Health Centers (FQHCs) or contracted as Medicaid Health Homes, but we wonder if policies promoting the adoption of medical home functions in these settings has ensured equal dissemination of functions across practice types [28, 51]. As of 2015, the year of the MOS, 20 states had established Medicaid Health Home programs [52]. Additional research is needed to look at state-level variation in order to determine if there are differences in states with and without Medicaid Health Home programs.

Our study is among the first to explore adoption of medical home practices among persons with and without PD. While it is encouraging that there is no difference in adoption of the medical home practices, future research should investigate if adoption of these practices is associated with improvements in access, satisfaction, utilization, and health outcomes for persons with PD.

### Limitations

There are a number of limitations to our study. The MOS survey was first fielded in 2015, thus there is no prior data available for comparison. The MOS only surveyed practices where the MEPS participants participant received the majority of their office-based care. The survey did not assess whether participants received care from any other primary care or specialty practices and the medical home functionality of those practices, including mental health centers. Community mental health clinics are actively adding medical home functions and seeking PCMH certification [53]. We did control for chronic illnesses as participants with chronic illnesses are more likely to receive care at multiple practices and have exposure to different levels of medical home functionality. Additionally, the MOS survey only includes MEPS participants who had a visit with a usual care provider in the previous twelve months. As a result, we can only generalize these findings to adults with and without PD that have a usual source of primary care.

The MOS respondents varied between the practices and the respondents may not have known all aspects of care delivery [34]. The MOS questioned the practices on how they generally deliver care to their patients; they did not ask about the care patterns of the affiliated MEPS participant (i.e. the practices were asked if they used case managers to coordinate care, not if the MEPS participant had their care coordinated by a case manager). The MOS did not offer definitions of the medical home functions to the survey respondents, which could have resulted in variability in interpretation. One practice could have interpreted routine use of the electronic records system to exchange secure messages with patients as a few times per week, while other practices could have interpreted this question as multiple times per day. Future studies should be cautious of limitations of this data set when trying to draw conclusions regarding the treatment effects of the medical home functions.

While the MOS asked the practices about their participation is ACOs and capitated contracts, it did not ask about participation in other programs designed to transform primary care, including the Medicaid Health Home [54, 55], the Federal Qualified Health Center Advanced Primary Care Practice Demonstration [51], private health plan PCMH programs [56, 57], or pay-for-performance programs [58]. Payment reform is a key driver in changing the way that primary care is delivered and we believe that future surveys should include additional questions regarding all alternative payment models and pay-for-performance programs that practices participate in.

The MOS did not survey practices about behavioral health integration. Behavioral health integration, systematic communication and coordination across behavioral health and general medical providers, is an essential component of providing comprehensive patient-centered care primary care to adults with PD [59]. Behavioral health integration is working to reduce the silos between primary care and mental health providers [60]. Patients with depression and anxiety that receive care from behavioral health integrated primary care practices have better outcomes, better medication compliance, and higher rates of satisfaction [61]. A 2014 survey of primary care practices that received PCMH certification found that 42% reported having a behavioral health clinician, psychiatrist, psychologist, social worker, or substance abuse counselor on site as part of the practice staff [62]. It will be important for the future MOS to include measures specifically related to behavioral health integration to track the dissemination of this critical medical home function.

## Conclusion

The way that primary care delivered is undergoing major changes and we are glad to see that adults with mental health issues are not being left behind. Medical home functions provide a structure for reducing cognitive overload and exhaustion, strengthening the patient-clinician relationship, promoting patient-provider communication about health, and facilitating patient involvement in their own care [63]. Ongoing policy initiatives, such as the Accountable Care Organization, the Comprehensive Primary Care model, and value-based financing model, are also needed and should be implemented to truly transform their practice into a medical home. Due to the complex needs of adults with mental health issues, the delivery of primary care through medical home models has significant potential to improve the health outcomes of this population. We expect that adoption of these medical home functions as well as behavioral health integration will continue to grow among primary care practices that care for this patient population.

## Abbreviations

ACO: Accountable Care Organization
AHRQ: Agency for Healthcare Research and Quality
ED: Emergency Department
EHR: Electronic Health Record
EMR: Electronic Medical Record
K6: Kessler Psychological distress screening assessment
MACRA: Medicare Access and CHIP Reauthorization Act
MEPS: Medical Expenditure Panel Survey
MOS: Medical Organizations Survey
PCMH: Patient-Centered Medical Home
PD: Psychological distress
PPACA: Patient Protection and Affordable Care Act
SMI: Severe Mental Illness
